# Beneficial Effect of Fluoxetine and Sertraline on Chronic Stress-Induced Tumor Growth and Cell Dissemination in a Mouse Model of Lymphoma: Crucial Role of Antitumor Immunity

**DOI:** 10.3389/fimmu.2018.01341

**Published:** 2018-06-19

**Authors:** María Emilia Di Rosso, Helena Andrea Sterle, Graciela Alicia Cremaschi, Ana María Genaro

**Affiliations:** ^1^Instituto de Investigaciones Biomédicas (BIOMED), Consejo Nacional de Investigaciones Científicas y Técnicas (CONICET)—Universidad Católica Argentina (UCA), Ciudad de Buenos Aires, Argentina; ^2^Departamento de Farmacología, Facultad de Medicina, Universidad de Buenos Aires (UBA), Ciudad de Buenos Aires, Argentina

**Keywords:** chronic stress, antitumor immunity, fluoxetine, sertraline, tumor invasion, lymphoma

## Abstract

Clinical data and experimental studies have suggested a relationship between psychosocial factors and cancer prognosis. Both, stress effects on the immune system and on tumor biology were analyzed independently. However, there are few studies regarding the stress influence on the interplay between the immune system and tumor biology. Moreover, antidepressants have been used in patients with cancer to alleviate mood disorders. Nevertheless, there is contradictory evidence about their action on cancer prognosis. In this context, we investigated the effect of chronic stress on tumor progression taking into account both its influence on the immune system and on tumor biology. Furthermore, we analyzed the action of selective serotonin reuptake inhibitors, fluoxetine and sertraline, in these effects. For this purpose, C57BL/6J mice submitted or not to a chronic stress model and treated or not with fluoxetine or sertraline were subcutaneously inoculated with EL4 cells to develop solid tumors. Our results indicated that chronic stress leads to an increase in both tumor growth and tumor cell dissemination. The analysis of cell cycle regulatory proteins showed that stress induced an increase in the mRNA levels of cyclins A2, D1, and D3 and a decrease in mRNA levels of cell cycle inhibitors p15, p16, p21, p27, stimulating cell cycle progression. Moreover, an augment of mRNA levels of metalloproteases (MMP-2 and MMP-9), a decrease of inhibitors of metalloproteases mRNA levels (TIMP 1, 2, and 3), and an increase in migration ability were found in tumors from stressed animals. In addition, a significant decrease of antitumor immune response in animals under stress was found. Adoptive lymphoid cell transfer experiments indicated that the reduced immune response in stressed animals influenced both the tumor growth and the metastatic capacity of tumor cells. Finally, we found an important beneficious effect of fluoxetine or sertraline treatment on cancer progression. Our results emphasize the crucial role of the immune system in tumor progression under stress situations. Although a direct effect of stress and drug treatment on tumor biology could not be ruled out, the beneficial effect of fluoxetine and sertraline appears to be mainly due to a restoration of antitumor immune response.

## Introduction

Nowadays, stress affects thousands of people around the world. Stress is defined as a critical, real, or apparent, situation that represents a challenge for homeostasis. To restore this state, a coordinated adaptive response is triggered. Stress mediators involve not only catecholamines and glucocorticoids (the characteristic neuroendocrine hormones of the stress response) but also several other neurotransmitters, cytokines, and growth factors (1). It is important to note that although stress response is an essential survival mechanism, when it is prolonged over time, may affect endocrine, immunological, and behavioral function (1). In particular, epidemiological studies indicate that chronic stress might constitute a risk factor for cancer onset and progression (2, 3).

The role of psychosocial factors in cancer initiation is ambiguous. However, the influence of stress on cancer progression has been demonstrated. Both clinical and experimental studies have shown that the mechanisms involved in stress response are capable of influencing processes related to cancer progression (4–6). Animal models that mimic the pattern of human disease have been used to understand the impact of stress on cancer and other pathologies. These studies put the main focus on the neuroendocrine modulation of the immune response to tumor cells (2, 7–10). Moreover, the direct effect of stress mediators on the proliferation and aggressive behavior of tumor cells, independently of the influence on the immune system, has been analyzed. The group of Sood demonstrated that chronic stress increase catecholamine levels in tumors that in turn promote metastasis of breast (11) and ovarian (12) carcinomas. In addition, in many experimental models, the biological consequences of stress have been shown to be reverted by β-adrenergic blockers (11). In addition, it was proposed that norepinephrine also promotes resistance to anoikis, inhibits apoptosis, and increases chemoresistance (13) of tumor cells. Nevertheless, in general, these studies were developed in athymic nude or SCID mice and analyze the influence of stress on tumor invasion and metastasis independently of the action of immune system (14).

In addition, selective serotonin reuptake inhibitors (SSRIs), as fluoxetine and sertraline, are frequently prescribed for the treatment of stress-associated disorders, such as depression, obsessive–compulsive disorder, panic attack, and bulimia nervosa. The use of antidepressants has been related to immune alterations. Nevertheless, conflictive data have been reported regarding the impact of fluoxetine on the immune system and cancer prognosis (15). In a previous report, we showed that fluoxetine reverts the effect of stress on T helper immunity through compensatory and/or specific mechanisms (16). In addition, fluoxetine was able to enhance the apoptosis/proliferation balance of lymphoma cells and increase T cell immunity in tumor-bearing mice (17).

In this context, the objective of this study was to investigate the effect of chronic stress on tumor progression taking into account both its influence on the immune system and its action on tumor biology. Moreover, we aimed to analyze the influence of two SSRIs, fluoxetine and sertraline, in these effects. For this purpose, we used EL4 T cell lymphoma cells growing as a solid tumor in C57BL/6J mice submitted or not to a variable stress model and treated or not with fluoxetine or sertraline. Our results indicate that tumor growth and metastases are affected by psychological stress. Cellular adoptive transfer approach pointed out that changes in tumor biology were predominantly the result of the influence of stress on the immune function. In addition, treatment with the SSRIs, fluoxetine and sertraline, prevented these effects. These findings strengthen the clinical research about the beneficial effects of the SSRIs prescription in cancer patients.

## Materials and Methods

### Cell Line and Culture Condition

The tumor cell line EL4 was obtained from American Type Culture Collection (ATCC, Manassas, VA, USA; Catalog Number TIB-39). Cells were cultured in RPMI 1640 with 10% fetal bovine serum (GIBCO). EL4 cell line was established from a lymphoma induced in a C57BL/6J mouse by 9,10-dimethyl-1,2-benzanthracene (18). Cells were cultured at an optimal concentration (1–5 × 10^5^ cells/ml) in RPMI-1640 medium supplemented with 10% v/v fetal bovine serum, 2 mmol/l glutamine, and 100 mg/ml streptomycin (all from Life Technologies), at 37°C in 5% CO_2_ atmosphere, as previously described (19).

### Animals

Inbred female C57BL/6J (H-2^b^) mice, 2–3 months old, were bred and kept at the Instituto de Investigaciones Biomédicas (BIOMED, CONICET-UCA, Buenos Aires, Argentina). Animals were cared for and sacrificed according to the rules of the “Guide for the Care and Use of Laboratory Animals” (NIH) (revision 2011) and to the EC Directive 86/609/EEC (revision 2010). The experimental protocol was also approved by the local Institutional Committee for the Use and Care of Laboratory Animal rules (CICUAL, BIOMED, Argentina).

### Chronic Stress Model and SSRIs Administration Protocol

The chronic stress model used consists in the aleatory, intermittent, and unpredictable exposure to different stressors during 5 weeks in C57BL/6J mice. Briefly, animals were randomly and alternately exposed to one of the following stressors for the time indicated for each assay: restraint in well-ventilated tubes for 6 h (20), tail suspension for 5 min (21), forced swimming for 5 min (22), cold temperature exposure (4°C) for 2 h (23, 24), and 2 days of continuous overnight illumination (25).

To analyze the effect of SSRIs, mice were orally given 15 mg/kg/day of fluoxetine (Sigma-Aldrich) (26) or 20 mg/kg/day of sertraline (Sigma-Aldrich) (27), in a fresh solution prepared in the drinking water. The preparation of these solutions was performed taking into account the volume of water drunk daily by each mouse (5 ml) to reach the indicated dose.

### Lymphoma Model and Tumor Growth

C57BL/6J syngeneic animals, under different treatments, received subcutaneous injections of 3 × 10^5^ EL4 cells in 200 µl of phosphate-buffered solution (PBS) to generate a solid tumor. Tumor length and width were measured every day using calipers, and tumor volume was calculated as *V* = π/6 × length × width (7). With the exception of mice used for the spontaneous metastasis test, mice were euthanized by CO_2_ overexposure 14 days post tumor cell injection or when tumor reached the maximum volume allowed by ethical standards (Guidelines for Endpoints in Animal Study Proposals, NIH).

### Quantitative Real-Time Reverse Transcription Polymerase Chain Reaction (qRT-PCR)

After 14 days of tumor injection, animals were sacrificed, solid tumors were dissected and instantly homogenized in Tri-Reagent (Genbiotech SRL) to isolate the RNA, following the manufacturer’s instructions. The RNA pellets were re-suspended in RNase-free water, and the RNA concentration was quantified by measuring the absorbance at 260 nm in a nanodrop (Nanodrop ND-1000, Thermo Fisher Scientific Inc.). The total RNA was used as a template to generate first-strand cDNA synthesis using the M-MLV Reverse Transcriptase (Invitrogen), random primers (Invitrogen), and dNTPs (Invitrogen). The cDNA amounts present in each sample were determined by a 7500 Real-Time PCR System (Applied Biosystems) using the KAPA SYBR^®^ FAST qPCR Kit Master Mix (2×) Universal (Kapa Biosystems) and following the manufacturer’s instructions. Each RT-PCR quantification experiment was performed in duplicate. To verify that the SYBR Green dye detected only one PCR product, all the reactions were subjected to a heat dissociation protocol following the final cycle of PCR. The sequences of mouse-specific primers, the annealing temperature, and the amplicon size are provided in Table 1. The primer sequences (Biodynamics SRL), shown in Table 1, were designed using the Primer Express Software version 3.0 (Applied Biosystems). To determine the target gene mRNA expression, the comparative cycle threshold (Ct) method was used (28). An average Ct value was calculated from the duplicate reactions and normalized to the expression of β_2_-microglobulin, and the 2(−ΔΔCt) value was calculated. It is important to note that similar results were obtained using cyclophilin or glucose-6-phosphate-dehydrogenase (G6PDH) mRNA expression levels as housekeeping (data not shown) (29).

**Table 1:** Primers sequences for quantitative real-time reverse transcription polymerase chain reaction.

Gene	Accession no.	Sequences	Amplicon size (pb)	Annealing T (°C)
Cyclin A2	NM_009828.2	Fw: 5′-GGCCAGCTGAGCTTAAAGAAAC-3′ Rv: 5′-CGGGTAAAGAGACAGCTGCAT-3′	69	61
Cyclin D1	NM_007631.2	Fw: 5′-CCAAAACCATTCCATTTCAAAG-3′ Rv: 5′-CCAACACACACCAGCAACACT-3′	68	61
Cyclin D3	NM_007632.2	Fw: 5′-TGCGTGCAAAAGGAGATCAA-3′ Rv: 5′-TCACACACCTCCAGCATCCA-3′	68	60
p15/INK4B	NM_007670.4	Fw: 5′-TGGGAAACCTGGAGAGTAGATGA-3′ Rv: 5′-GAATCCCCACACATGACAGTACA-3′	66	58
p16/INK4A	NM_009877.2	Fw: 5′-CTCAACTACGGTGCAGATTCGA-3′ Rv: 5′-CACCGGGCGGGAGAA-3′	57	58
p21/Cip1	NM_007669.5	Fw: 5′-TGTGGCTCCCTCCCTGTCT-3′ Rv: 5′-GCAGGGTGCTGTCCCTTCT-3′	63	58
p27/Kip1	NM_009875.4	Fw: 5′-CCTGGCTCTGCTCCATTTGA-3′ Rv: 5′-ACGGATGGAGCGCAAAAC-3′	71	58
MMP-2	NM_008610.3	Fw: 5′-TCTGGTGCTCCACCACATACAACT-3′ Rv: 5′-CTGCATTGCCACCCATGGTAAACA-3′	90	60
MMP-9	NM_013599.4	Fw: 5′-TGAACAAGGTGGACCATGAGGTGA-3′ Rv: 5′-TAGAGACTTGCACTGCACGGTTGA-3′	121	60
Timp-1	NM_001044384.1	Fw: 5′-GGTGTGCACAGTGTTTCCCTGTTT-3′ Rv: 5′-AAGCAAAGTGACGGCTCTGGTAGT-3′	119	60
Timp-2	NM_011594.3	Fw: 5′-TTTCTAGCCACACCAGGCAGATGA-3′ Rv: 5′-GGTTTGCTGGGAAGGCATTTGAGT-3′	112	60
Timp-3	NM_011595.2	Fw: 5′-ACCACTGCTTTGTCCAGGTGTTTG-3′ Rv: 5′-ATGGAAATGGTTGTGCCTTCTGCC-3′	145	64
β_2_-microglobulin	NM_009735.3	Fw: 5′-GCTATCCAGAAAACCCCTCAA-3′ Rv: 5′-CATGTCTCGATCCCAGTAGACGGT-3′	300	58
Cyclophilin B	NM_011149.2	Fw: 5′-CGAGTCGTCTTTGGACTCTTT-3′ Rv: 5′-GCCAAATCCTTTCTCTCCTGTA-3′	87	58
G6PDH	NM_008062.2	Fw: 5′-GAAGCTGCCAATGGATACTTAGA-3′ Rv: 5′-CCACCGTTCATTCTCCACATAG-3′	99	58

### Disaggregation of Solid Tumor

After 14 days of tumor injection, mice were sacrificed, and solid tumors were dissected. To obtain cells from solid tumors, a modification of conventional method of disaggregation by trypsinisation was used (30). Briefly, tumors were fragmented and were incubated at 37°C for 30 min with a solution containing 0.25% trypsin and 0.004% of DNAse in PBS in a relation of 10 ml per 1 ml of tissue. After incubation, the trypsin solution containing dissociated cells was collected into a sterile 50-ml centrifuge tube. Immediately, an equal volume of RPMI medium containing 10% FBS was added to inactivate the trypsin and protect the cells from continued proteolytic digestion. The cell suspension was centrifuged for 5 min at 400 *g* and re-suspended in culture medium. This procedure was repeated two times to obtain the optimal tissue disaggregation. Cell viability was checked by trypan blue exclusion test and settled to the desired concentration.

### Evaluation of Metastatic Properties of Tumor Cells

To analyze the metastatic properties of tumor cells, spontaneous and experimental metastasis assays were used (31). One group of solid tumor-bearing mice was used for spontaneous metastasis assessment. These mice were monitored every day and were euthanized when they exhibited characteristic of animals that are about to die such as signs of suffering, hypothermia, and slow locomotion. Animals were sacrificed at day 19 post EL4 cells subcutaneous injection, and the number of metastatic nodules in kidney and liver was determined. For the experimental metastasis tests, mice were inoculated through the tail vein either with 5 × 10^5^ EL4 cells or with solid tumor disaggregated cells from the different experimental groups. After 14 days, mice were killed, organs were removed, and metastatic nodules were counted.

### Migration Assay

Tumors from mice of different experimental groups were disaggregated as described in Section “Disaggregation of Solid Tumor” and 5 × 10^4^ cells of each tumor were re-suspended in RPMI culture medium without FBS, seeded into the top well of a transwell chamber with 8.0-µm pores (Jet Biofil), and allowed to migrate toward medium containing 10% of FBS for 24 h. Cells in the upper and in the lower compartment were counted using a Neubauer chamber. Cell migration is presented as percentage of total cell count for each sample (32).

### Natural Killer Activity Assay

YAC-1 cells were acquired from ATCC (Catalog number TIB-160). Cells were maintained in supplemented medium as described for EL4 cells. Specific cytotoxic activity against tumor cells was determined according to the just another method (JAM method) as previously reported (7). Briefly, YAC-1 cells were cultured in the presence of 5 mCi [^3^H]-thymidine for 16 h. Cell suspensions from spleens of mice from different groups were obtained. Briefly, spleens were removed and disrupted through a 1-mm metal mesh, and the cell suspensions were filtered through a 10-lm nylon mesh. The suspensions were depleted of red blood and dead cells using a lysis buffer (NH_4_Cl 8.29 g, KHCO_3_ 1 g, EDTA-2Na 37.2 mg, diluted in distilled water, at pH = 7.4) for 2 min. After three washes in PBS, cells were re-suspended in PBS at final concentration. Cell viability was assessed by trypan blue exclusion assay. A target:effector ratio 1:50 was seeded in 96-well plates at a final volume of 200 µl, and incubated for 3.5 h at 37°C in a 5% CO_2_ atmosphere. [^3^H]-Thymidine incorporation was measured by scintillation counting after retention over GF/C glass-fiber filters (Whatman). NK activity was calculated as 100 × (SR − ER)/SR, where SR is the spontaneous release and ER is the experimental release.

### Cytotoxic Activity Assays

Specific cytotoxic activity against tumor cells was evaluated according to the JAM test (7) as previously described. Briefly, EL4 labeled overnight with 5 mCi [^3^H]-thymidine were co-cultured with spleen cell suspensions from tumor-bearing mice from the different treatments at a target:effector ratio of 1:15 for 3.5 h. The percentages of cytotoxic activity were calculated as the following relation: cytotoxic activity of T lymphocytes = 100 × (SR − ER)/SR, where SR is the spontaneous release and ER is the experimental release.

### Total-Body γ-Irradiation and Lymphoid Cell Transplantation

Two-month-old C57BL/6J mice were placed individually into 1-mm thick, rectangular plastic boxes (30 mm × 30 mm × 60 mm) with holes to allow free exchange of air. Mice were exposed to a single dose of 2 Gy applied to the total body at a rate of 0.8 Gy/min. Gamma-irradiation was performed using a vertical beam containing ^137^Cs source (Cebirsa SA, Buenos Aires, Argentina). This procedure provokes a lymphocyte depression near 80% (33).

On the following day, mice were transplanted with lymphoid cells that were obtained from mice of the different experimental groups. For this purpose, mice were sacrificed and lymph nodes (axillary, inguinal, and mesenteric) were obtained and disaggregated through a 1-mm metal mesh, and the cell suspension was filtered through a 10-µm nylon mesh. After three washes in PBS, cells were re-suspended at final concentration (7). A volume of 0.1 ml of cell suspension containing 8 × 10^6^ lymphoid cells was transplanted into the recipients *via* tail vein injection.

### Depletion of Immune Cells in Disaggregated Tumors

After 14 days of tumor injection, mice were sacrificed, and solid tumors were dissected. To purify tumor cells, an immune cell complement depletion protocol was used (34). Noteworthy, EL4 cells are CD4 and CD8 negative, so lymphoma cells are not affected by complement-dependent antibody-mediated lysis. Briefly, tumors were disaggregated as explained in Section “Disaggregation of Solid Tumor.” The cell suspension was adjusted to 2 × 10^7^ cells/ml and anti-mouse CD8a, CD4, and F4/80 (BD Biosciences) were added at a proper predetermined dilution. Then, cells were incubated for 30 min at 4°C and centrifuged at 400 *g* for 5 min. The pellet was re-suspended in RPMI containing 10% of low toxicity rabbit complement, incubated for 1 h at 37°C mixing every 15 min, and centrifuged at 400 *g* for 5 min. The cell pellet was then re-suspended either in RPMI-1640 culture for migration assay or in Tri-Reagent (Genbiotech SRL) to isolate the RNA. To confirm the effectiveness of the depletion protocol, a flow cytometric analysis was performed, comparing tumor cell suspensions before and after depletion. Cell suspensions were incubated with CD4, CD8, and F40/80 conjugated antibodies. All three cell populations were reduced after treatment. Macrophages from 15.6 to 1.5%, CD8+ cells from 6.5 to 1.2%, and CD4+ cells 4.7 to 0.6%. Total immune cells from 26.8 to 3.3%.

### Statistical Analysis

Data were expressed as the mean ± SEM for each group. All the data were processed using STATISTICA software (StatSoft, Inc., Tulsa, OK, USA). The normality and homogeneity of variance for the dataset were tested using the Shapiro–Wilk test and Levene’s test, respectively. Growth tumor data were analyzed with repeated measures two-way ANOVA analysis with condition (control or stressed) and pharmacological treatments (vehicle, fluoxetine, and sertraline) as factors. Other data were analyzed by two-way ANOVA with condition and pharmacological treatment as factors. For experiments using immune cell-depleted suspensions, one-way ANOVA was used. In all cases, if ANOVA showed significant differences between groups, Fisher’s *post hoc* test was performed to determine significance level. Student’s *t*-test was used for two group comparisons. Non-parametric Mann–Whitney *U* test was carried out to compare the number of metastatic nodules found in control and stressed mice. The binomial distribution test for comparing two proportions was used to analyze the statistical significance of % mice with spontaneous metastasis. *p* < 0.05 was considered to indicate a statistically significant difference.

## Results

### Fluoxetine and Sertraline Are Able to Prevent the Promotion of EL4 Lymphoma Growth Induced by Chronic Stress

**Figure 1:**
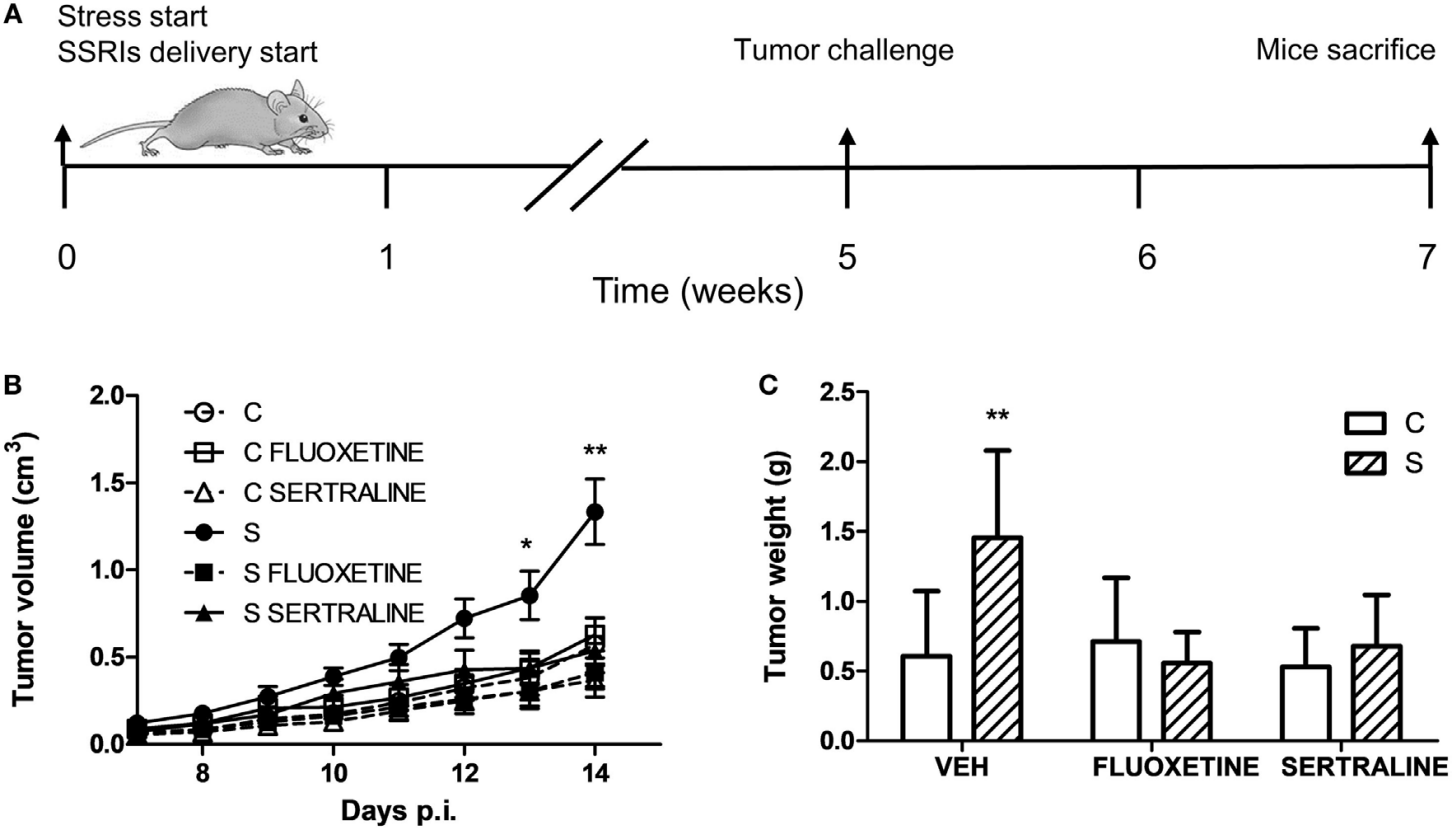
Effect of fluoxetine and sertraline on stress-induced tumor progression. **(A)** Schematic representation of experimental protocol. C57BL/6J mice were treated either with (S) or without (C) the chronic stress protocol, and with or without (VEH) fluoxetine or sertraline. Five weeks later, 3 × 10^6^ EL4 cells were subcutaneously injected to generate solid tumors. Two weeks post injection (p.i.), mice were sacrificed. **(B)** Time course of tumor volume for the different experimental groups. **(C)** Tumor weight at day 14 post EL4 cells injection. Values are expressed as mean ± SEM. *n* = 6 mice per group. *p < 0.05; **p < 0.01 respect to control mice.

To investigate the effect of chronic stress on tumor growth, 5-week-stressed and control mice were subcutaneously inoculated with EL4 syngeneic lymphoma cells to develop a solid tumor and tumor volume was determined every day. The stress protocol continued until the sacrifice of the mice. To analyze the effect of fluoxetine or sertraline treatment, drugs were dispensed orally simultaneously to stress exposure (see Figure 1A). A significant effect depending on time, stress exposure, and SSRIs treatment was found (*F*_14,210_ = 5.299; *p* < 0.001). As it can be seen in Figure 1B, data indicated that tumor growth was accelerated in chronically stressed mice respect to control animals after day 13. Interestingly, both fluoxetine and sertraline treatments counteract the stress effects on tumor growth. Accordingly, tumor weight at day 14 post EL4 cells injection was significant depending on stress exposure and SSRIs treatment (*F*_2,30_ = 4.460; *p* = 0.020) (Figure 1C).

**Figure 2:**
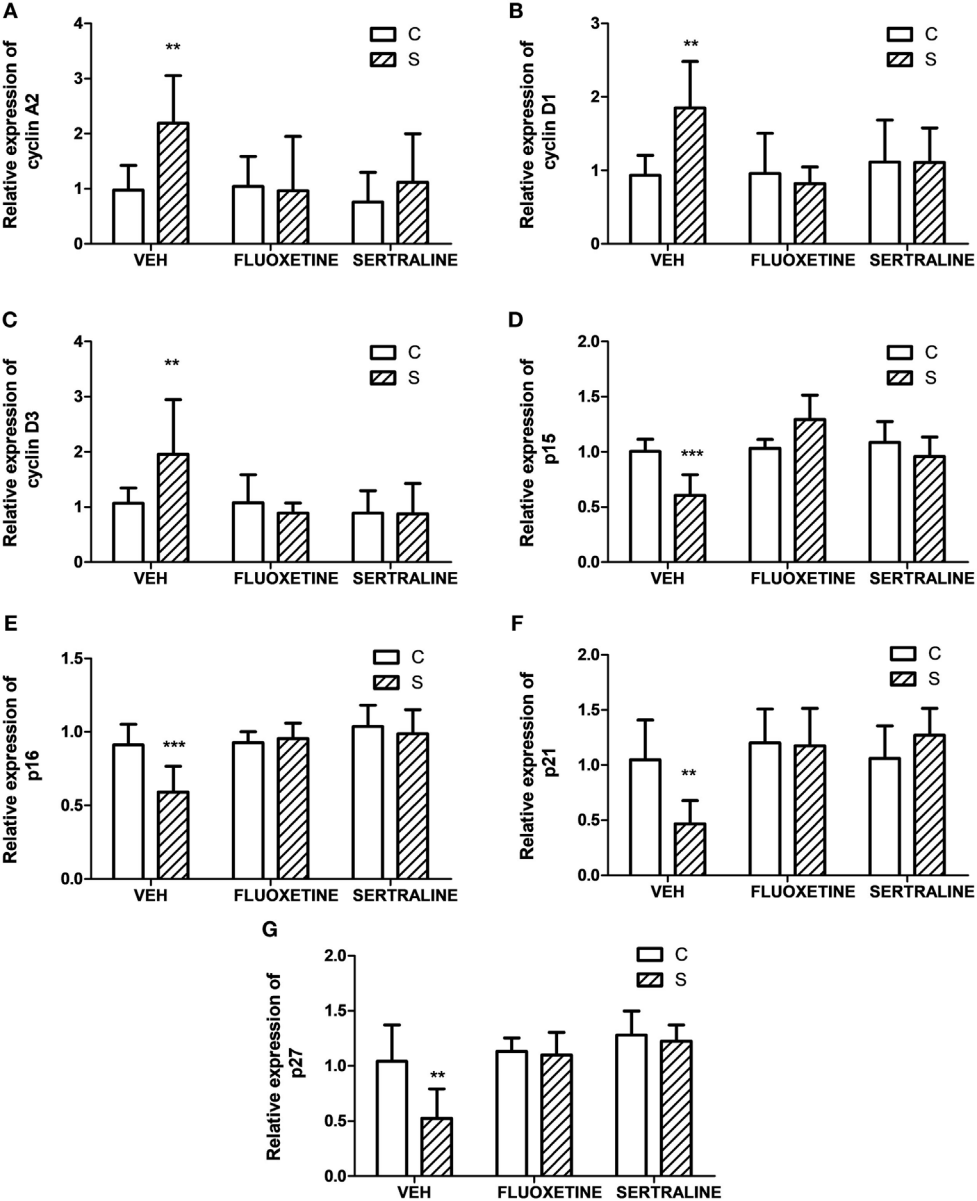
Effect of chronic stress on the expression of proteins associated with cell cycle progression. Action of fluoxetine or sertraline treatment. Quantitative real-time reverse transcription polymerase chain reaction was performed in cDNA obtained from solid tumors excised 14 days post EL4 cells inoculation. **(A)** Cyclin A2, **(B)** D1, **(C)** D3, CDKIs **(D)** p15/Ink4b, **(E)** p16/Ink4a, **(F)** p21/Cip1, and **(G)** p27/Kip1 mRNA relative expression was quantified using β_2_-microglobulin as housekeeper. Values are expressed as mean ± SEM. *n* = 6 mice per group. **p < 0.01, ***p < 0.001 respect to control mice.

To ascertain if proteins involved in the regulation and progression of cell cycle could be altered in parallel with tumor growth, we evaluated the tumor mRNA expression of A2, D1, and D3 cyclins and their inhibitors p15/Ink4b, p16/Ink4a, p21/Cip1, and p27/Kip1. Two-way ANOVA indicated that mRNA expression depended on stress exposure and SSRIs treatment (interaction stress × SSRIs, A2, *p* = 0.037; D1, *p* = 0.045; D3, *p* = 0.036; p15, *p* < 0.001; p16, *p* = 0.020; p21, *p* = 0.016; p27, *p* = 0.048). Results displayed in Figure 2 indicate that mRNA levels of cyclins A2, D1, and D3 were increased in tumors from animals under stress. In addition, their inhibitors were decreased in tumors from stressed animals. Moreover, both fluoxetine and sertraline treatments restored mRNA expression levels of these regulatory proteins to control values.

### Chronic Stress Increases the Tumor Invasion Capacity: Effect of Fluoxetine and Sertraline Administration

**Figure 3:**
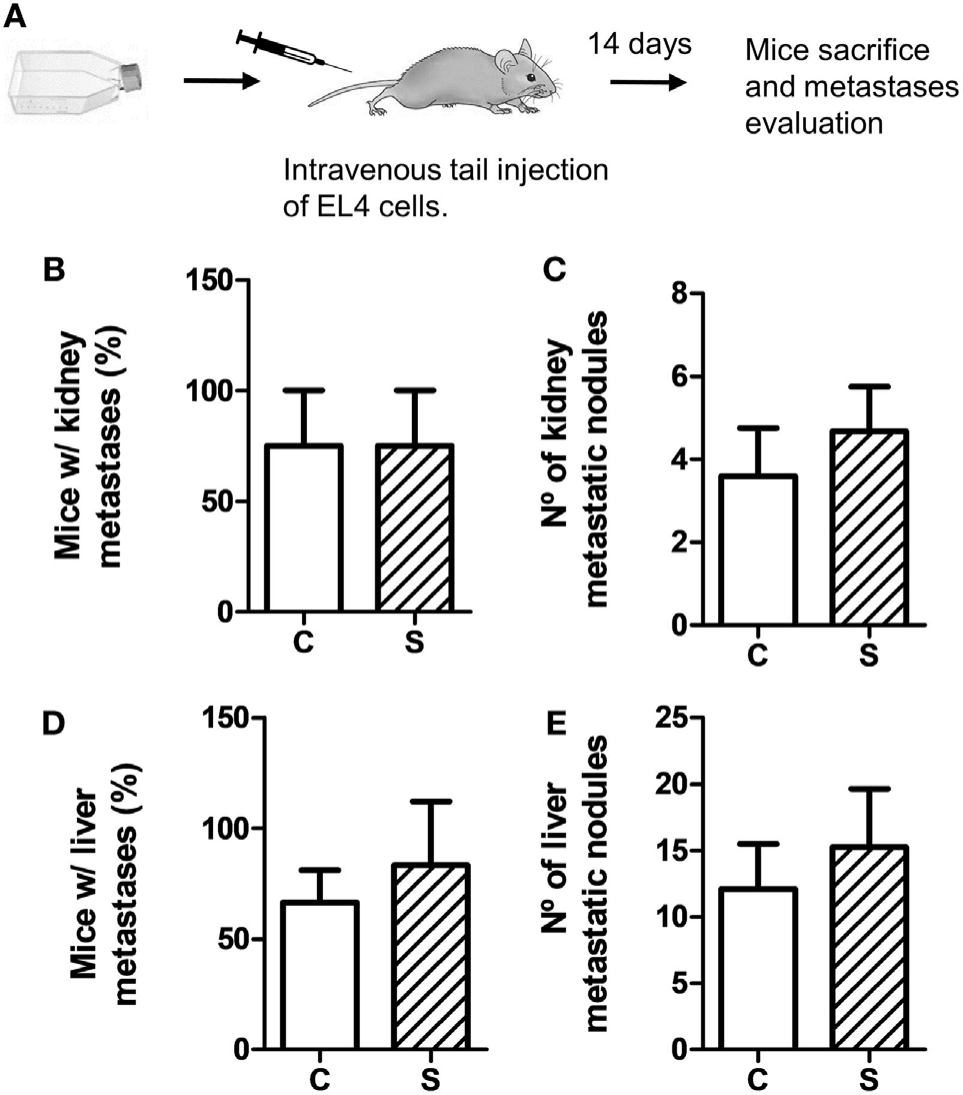
Experimental metastasis assay using EL4 cells. Experimental metastasis were evaluated 14 days after intravenous tail injection of 5 × 10^5^ EL4 cells in mice subjected (S) or not (C) to chronic stress treatment. **(A)** Schematic representation of experimental protocol. Percentage of mice with metastatic nodules in kidney **(B)** or liver **(D)** was calculated. Number of metastatic nodules in kidney **(C)** or liver **(E)** was assessed. Results are the mean ± SEM of three independent experiments (*n* = 4 mice per group).

**Figure 4:**
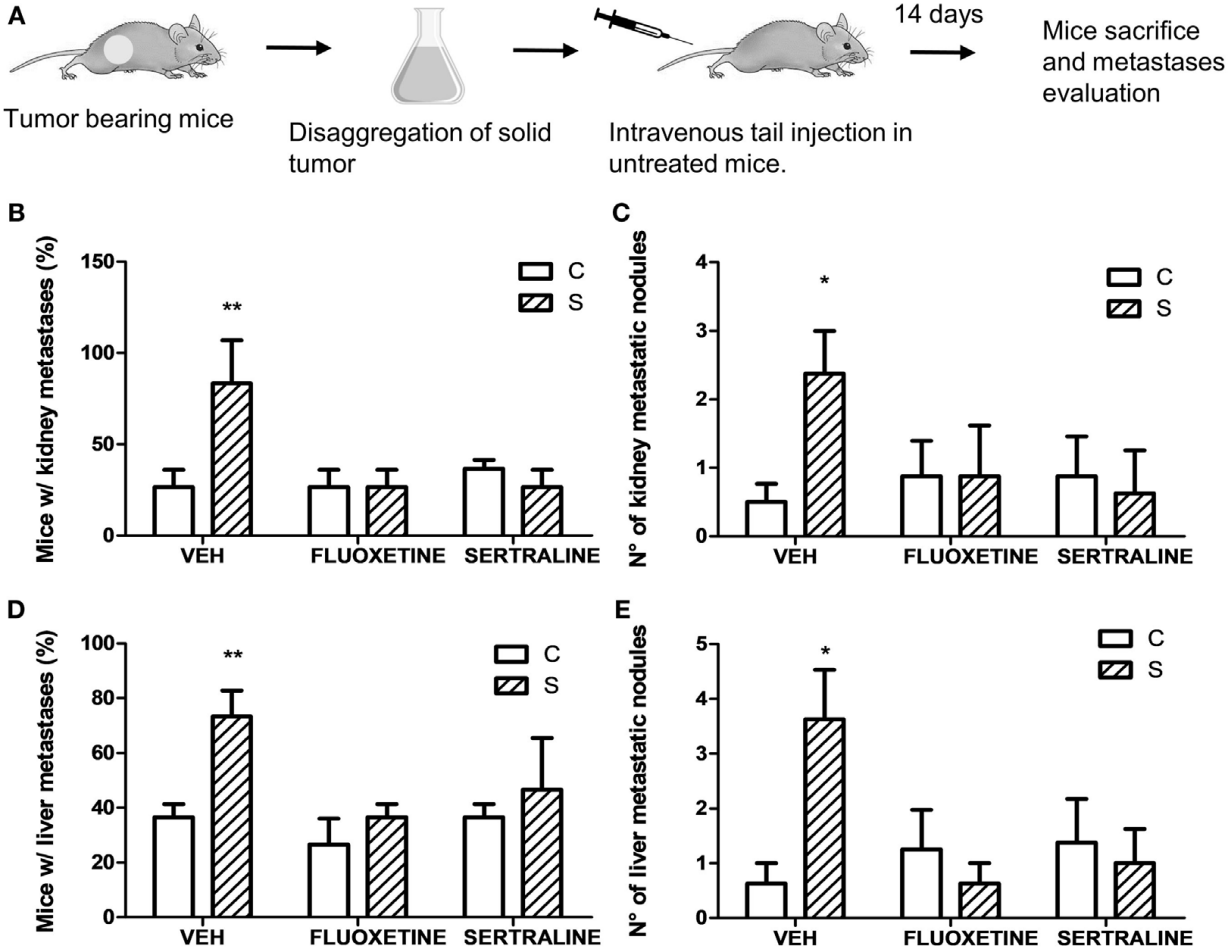
Experimental metastasis assay using cells from solid tumors of different experimental groups. 14 days post subcutaneous injection of EL4 cells in mice from different experimental groups, solid tumors were excised and disaggregated. 5 × 10^5^ cells were injected intravenously in untreated mice. Two weeks later, mice were sacrificed. **(A)** Schematic representation of experimental protocol. Percentage of mice with metastatic nodules in kidney **(B)** or liver **(D)** was calculated. Number of metastatic nodules in kidney **(C)** or liver **(E)** was assessed. Results are the mean ± SEM of two independent experiments (*n* = 5 and 4 mice per group). *p < 0.05, **p < 0.01 respect to control mice.

To evaluate metastatic dissemination capacity of tumor cells in different experimental groups, we performed experimental metastasis tests according to two experimental designs (see Figures 3A and 4A). In general, intravenous injection into the tail vein results in lung metastasis. However, it has been reported that EL4 cells mainly generate liver and kidney metastasis (35–37). First, we analyzed EL4 cell dissemination in control and stressed mice. For this procedure, EL4 cells cultured in standard conditions were injected in the tail vein (Figure 3). As it can be seen in Figure 3, the number of mice that presented metastatic nodules in the liver and kidney was not significantly different for control and stressed mice (liver: *t*_4_ = 0.894, *p* = 0.422; kidney: *t*_4_ = 0, *p* = 1). Moreover, Mann–Whitney *U* test revealed no significant differences in the number of metastatic nodules in these organs between both groups (liver: *U* = 62, *p* = 0.563; kidney: *U* = 59, *p* = 0.453).

Taking into account that stress was able to modify mRNA expression levels of proteins that regulate tumor growth, we analyzed the possibility that stress exposure could modify the dissemination capacity of tumor cells. For this purpose, solid tumors from different experimental groups were dissected and disaggregated to obtain cell suspensions. These cells were tail vein injected in untreated mice (Figure 4A). Results indicate that the incidence in the metastasis development depended on stress exposure and SSRIs treatment of the injected cells, in both kidney and liver (interaction stress × SSRIs: *F*_2,6_ = 8.30, *p* = 0.018; *F*_2,6_ = 8.03, *p* = 0.02, respectively). *Post hoc* analyses indicated that the percentage of mice with both kidney and liver metastasis was significantly higher in mice injected with cells from tumor of stressed mice (Figures 4B,D). Non-parametric analyses of the number of metastatic nodules found in liver and kidney, revealed a greater number of metastatic nodules in mice injected with cells from tumor of stressed mice respect to control mice (liver, *U* = 8.5, *p* = 0.013; kidney, *U* = 10.5, *p* = 0.024) (Figures 4C,E).

**Figure 5:**
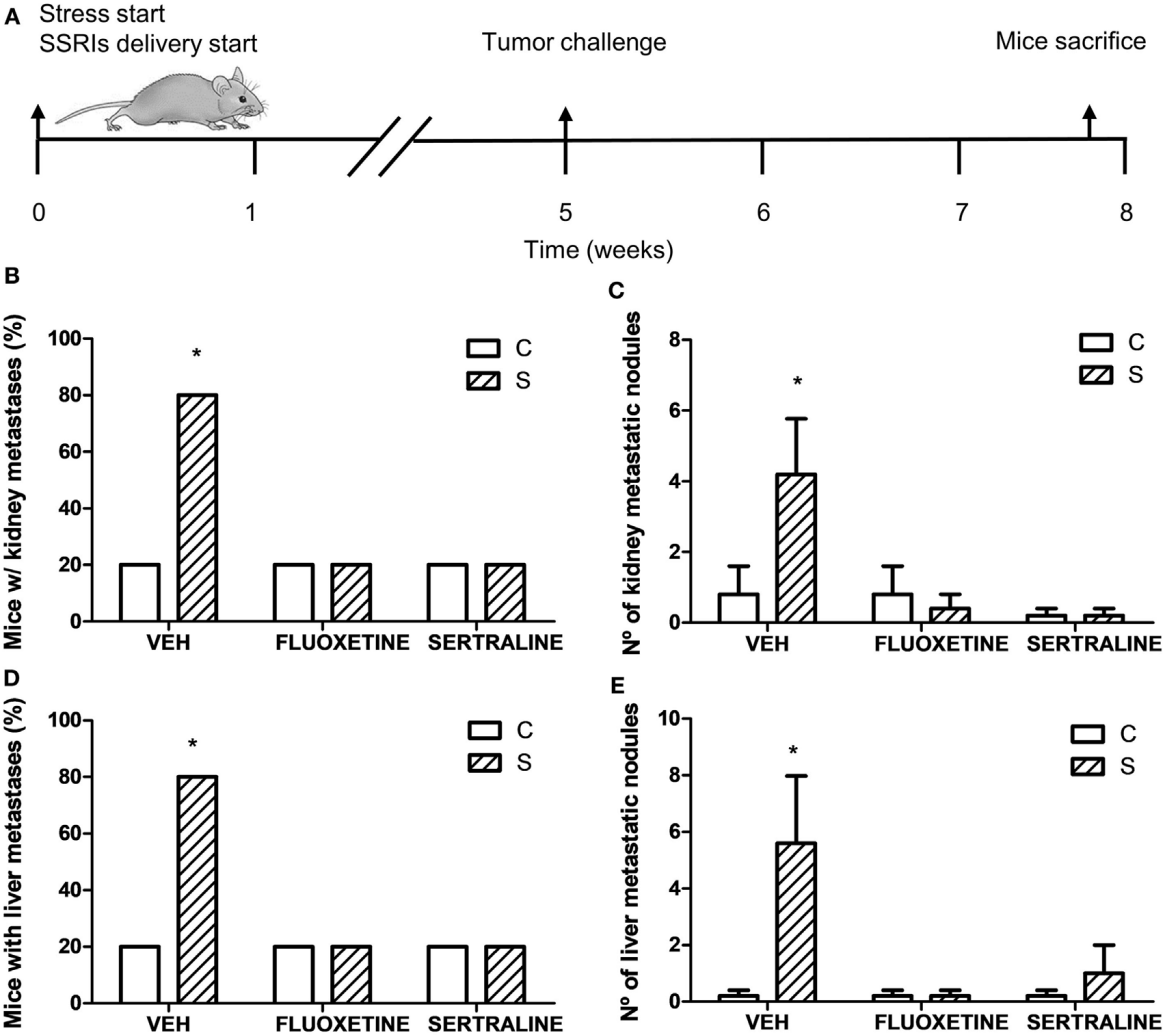
Spontaneous metastasis assay in mice from different experimental groups. **(A)** Schematic representation of experimental protocol. 19 days post subcutaneous injection of EL4 cells in mice from different experimental groups, mice were sacrificed and percentage of mice with metastatic nodules in kidney **(B)** or liver **(D)** was calculated. Also, number of metastatic nodules in kidney **(C)** or liver **(E)** was assessed. Values are expressed as mean ± SEM. *n* = 5 mice per group. *p < 0.05 respect to control mice.

Considering these results, we performed one experiment to determine spontaneous metastasis to evaluate the ability of cells to spread from a tumor implanted subcutaneously. For this purpose, mice were sacrificed 19 days after EL4 cells subcutaneous injection (Figure 5A). The binomial distribution test for comparing two proportions showed that the percentage of mice with metastasis was significantly higher in the stressed mice compared to control mice in both liver and kidney (*p* = 0.047) (Figures 5B,D). Mann–Whitney test showed a greater number of metastatic nodules in stressed mice respect to control group (liver, *U* = 3.5, *p* = 0.044; kidney, *U* = 4, *p* = 0.050) (Figures 5C,E). Interestingly, fluoxetine or sertraline treatment prevented these effects.

**Figure 6:**
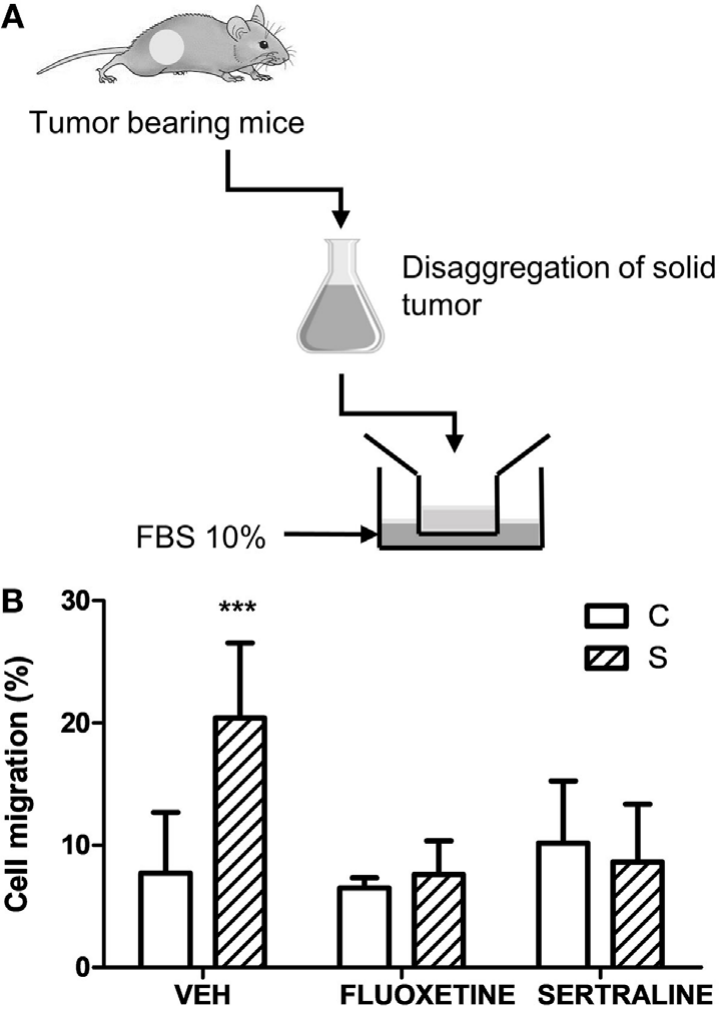
Effect of chronic stress and antidepressant treatment on tumor cells migration. **(A)** Schematic representation of the experimental protocol. 14 days post subcutaneous injection of EL4 cells in mice from different experimental groups, solid tumors were excised and disaggregated. 5 × 10^4^ cells were seeded in the upper well of transwell migration chamber and RPMI with 10% FBS as attractant. After 24 h, cells in the upper and in the lower chamber were counted. **(B)** Cell migration is presented as percentage of total cell count for each sample. Values are expressed as mean ± SEM. *n* = 5 mice per group. ***p < 0.001 respect to control mice.

In accordance with these results, two-way ANOVA of transwell migration assay data showed that the percentage of migration depended on stress exposure and SSRIs treatment (interaction stress × SSRIs, *F*_2,23_ = 7.143, *p* = 0.004). *Post hoc* analysis indicated that cells from tumors of stressed animals have a major migration capacity in a transwell chamber using FBS as attractant. As expected, fluoxetine or sertraline treatment eliminated this effect (Figure 6).

**Figure 7:**
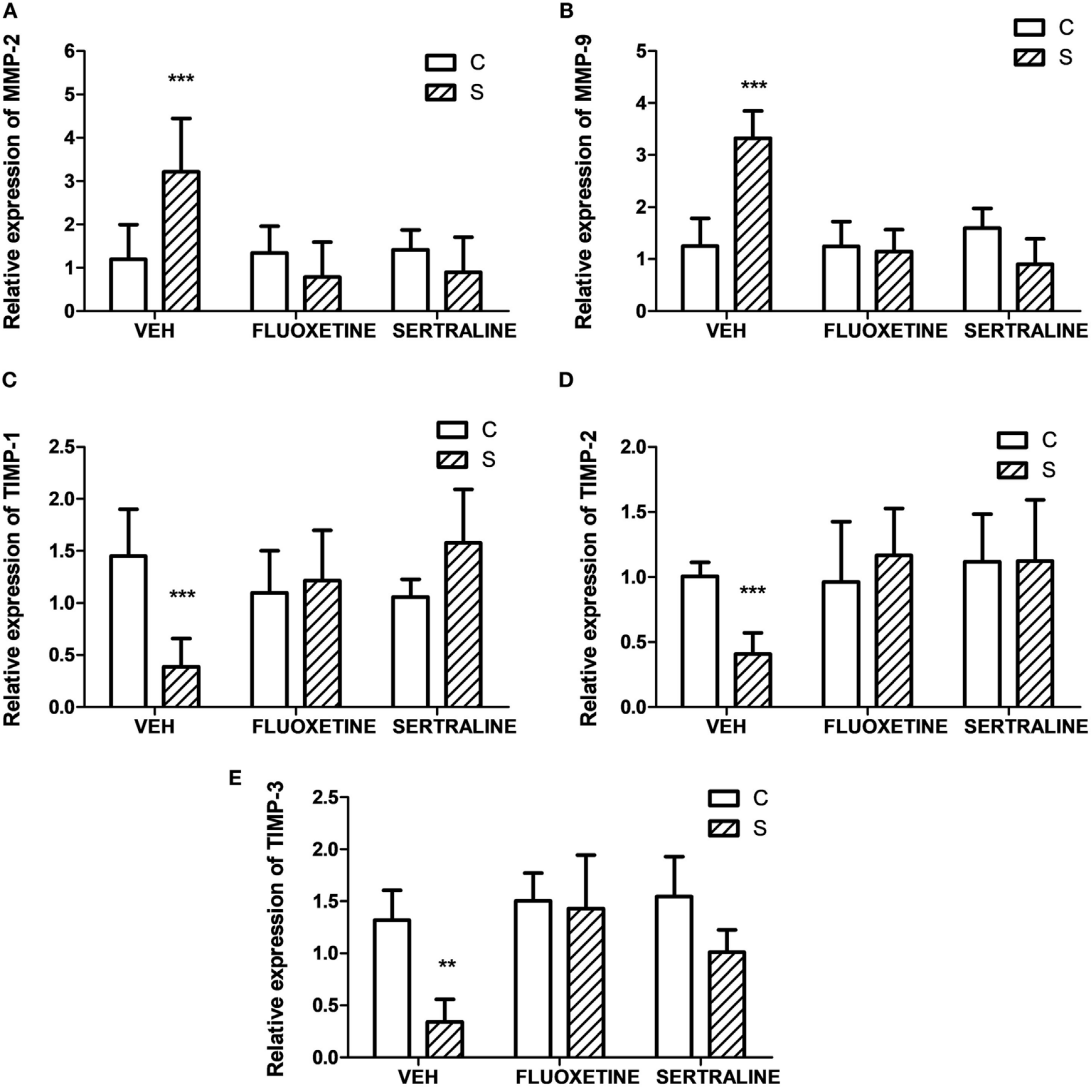
Effect of chronic stress on the expression of proteins associated with cell invasion. Action of fluoxetine and sertraline. Quantitative real-time reverse transcription polymerase chain reaction was performed in cDNA obtained from solid tumors excised 14 days post EL4 cells inoculation. **(A)** MMP-2, **(B)** MMP-9 and their inhibitors **(C)** TIMP-1, **(D)** TIMP-2, and **(E)** TIMP-3 mRNA relative expression was quantified using β_2_-microglobulin as housekeeper. Values are expressed as mean ± SEM. *n* = 6 mice per group. **p < 0.01; ***p < 0.001 respect to control mice.

Altogether, these findings indicate that stress-induced alterations in the biological behavior of tumors, and fluoxetine and sertraline were able to prevent these changes. In this context, invasion-related genes such as metalloprotease 2 (MMP2) and MMP9, and their inhibitors (TIMP 1, 2, and 3) were determined. As it can be seen in Figure 7, chronic stress significantly upregulated the expression of MMP2 and 9 in tumors and downregulated the expression of TIMPs. Also, fluoxetine and sertraline impeded these effects (interaction stress × SSRIs: MMP-2, *p* = 8.079; MMP-9, *p* < 0.001; TIMP-1, *p* < 0.001; TIMP-2, *p* = 0.005; TIMP-3, *p* = 0.046).

### Fluoxetine or Sertraline Administration Prevents the Decrease of Antitumor Immune Response Induced by Chronic Stress

**Figure 8:**
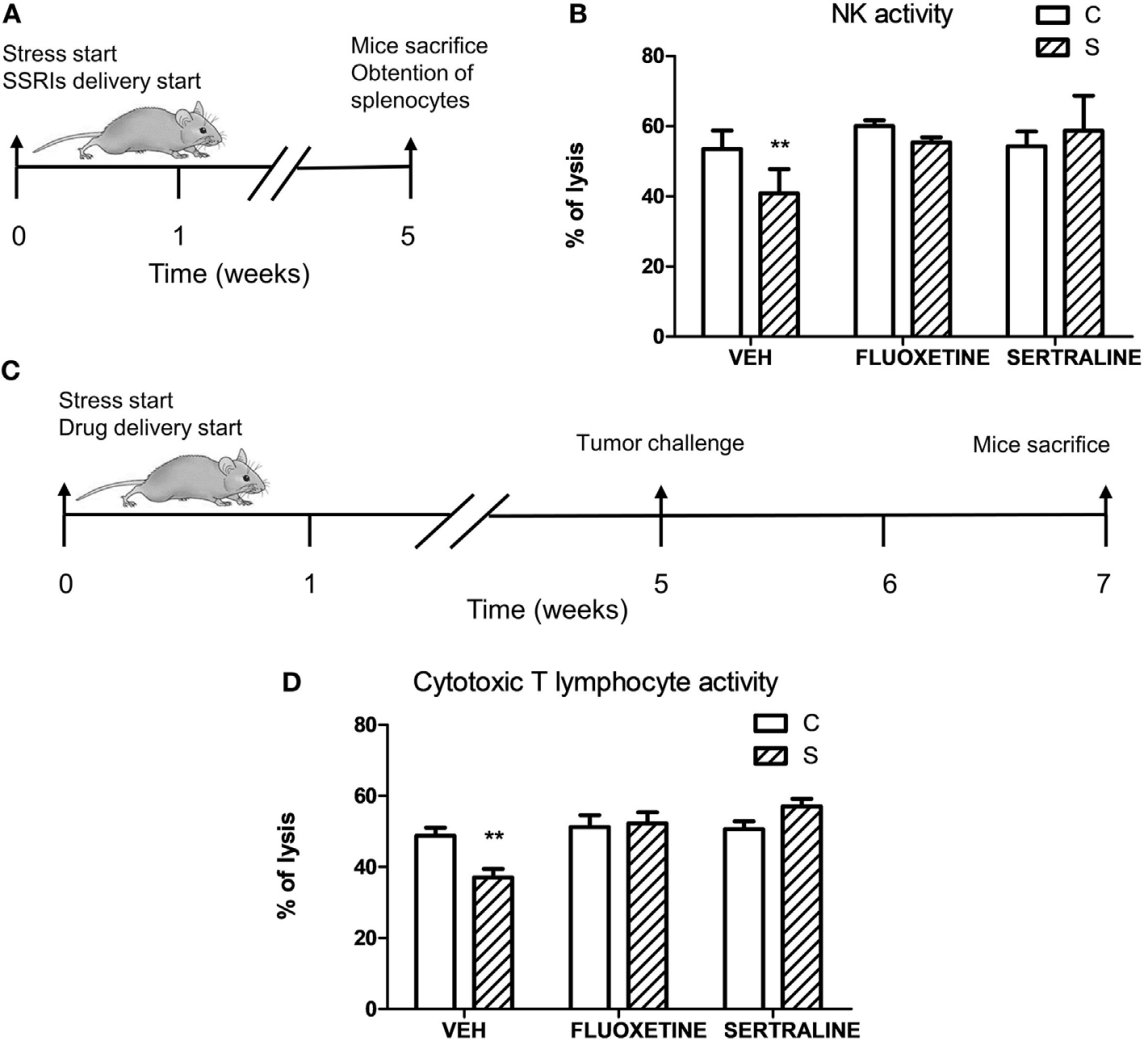
Influence of chronic stress and antidepressant treatment on antitumor immunity. **(A)** Schematic representation of experimental protocol. Mice were treated either with (S) or without (C) the chronic stress protocol, and with or without (VEH) fluoxetine or sertraline. Five weeks later, mice were sacrificed. **(B)** Splenocytes of mice from different experimental groups were co-incubated with YAC-1 cells labeled with [3 H]-thymidine at a target:effector ratio 1:50, cultured for 3.5 h and harvested. *n* = 4 mice per group. **(C)** Schematic representation of experimental protocol. C57BL/6J mice were treated either with (S) or without (C) the chronic stress protocol, and with or without (VEH) fluoxetine or sertraline. Five weeks later, 3 × 10^6^ EL4 cells were subcutaneously injected to generate solid tumors. Two weeks post injection (p.i.), mice were sacrificed and spleen cell suspensions were obtained. **(D)** Specific cytotoxic activity against tumor cells was evaluated co-culturing spleen cells suspensions from tumor-bearing mice and labeled EL4 cells with [3H]-thymidine at a target:effector ratio 1:15 for 3.5 h and harvesting. Percentages of NK or cytotoxic activity were calculated as 100 × (SR − ER)/SR, where SR is the spontaneous release and ER is the experimental release. *n* = 7 mice per group. Values are expressed as mean ± SEM. **p < 0.01 respect to control mice.

To investigate if stress exposure and drug treatment affect antitumor immune responses, we evaluated the NK activity in mice that were not exposed to the tumor challenge (Figure 8A) and the specific cytotoxicity against EL4 cells in tumor-bearing mice (Figure 8C). Two-way ANOVA of NK activity data revealed that the % of lysis of YAC-1 cells depended on stress exposure and SSRIs treatment (interaction stress × SSRIs, *F*_2,16_ = 4.008, *p* = 0.039). As it is shown in Figure 8B, splenocytes from stressed mice showed a decreased cytotoxic activity mediated by NK cells. This impaired NK activity was prevented by fluoxetine and sertraline administration. Two-way ANOVA of the specific cytotoxicity assay showed that immune cells from spleens of tumor-bearing animals of the different experimental groups were able to lyse the EL4 cells depending on stress exposure and SSRIs treatment (interaction stress × SSRIs, *F*_2,36_ = 6.354; *p* = 0.004). As it can be seen in Figure 8D, the percentage of EL4 cell lysis was significantly lower when EL4 cells were incubated with splenocytes from stressed mice compared to control mice. Once again, fluoxetine and sertraline treatments counteracted this effect.

Finally, to determine if alteration of antitumor immune responses could be involved in the promotion of tumor growth and tumor invasion capacity induced by stress exposure, adoptive transfer experiments were performed.

**Figure 9:**
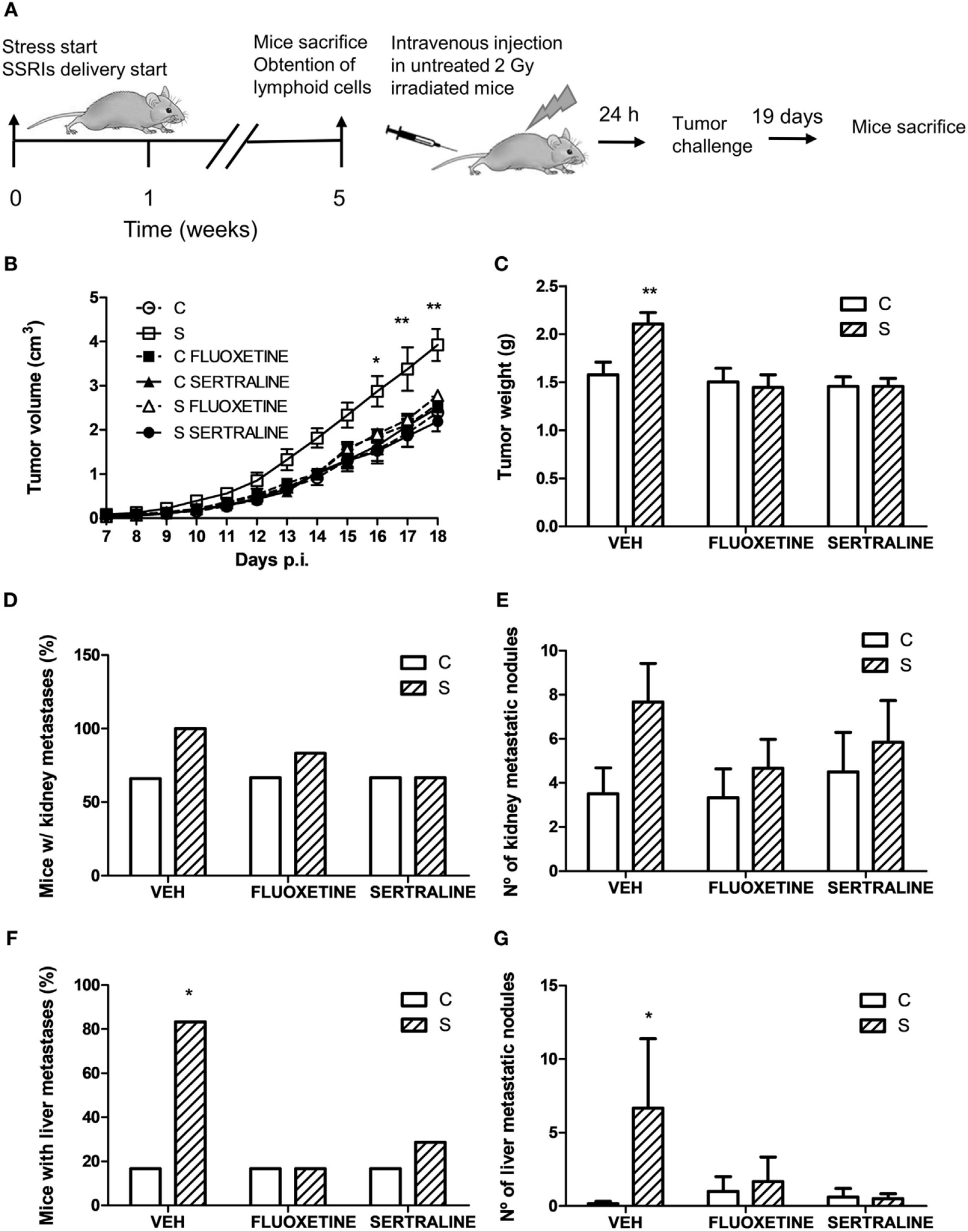
Evaluation of antitumor immunity role in tumor biological behavior using adoptive cell transfer strategy. **(A)** Schematic representation of experimental protocol. Mice were treated either with (S) or without (C) the chronic stress protocol, and with or without (VEH) fluoxetine or sertraline. Five weeks later, mice were sacrificed and lymphoid cells from different experimental groups were tail vein injected in untreated irradiated mice. After 24 h, EL4 cells were subcutaneously injected to generate solid tumors. 19 days post subcutaneous injection, mice were sacrificed, and spontaneous metastasis was evaluated. **(B)** Time course of tumor volume for the different experimental groups. **(C)** Tumor weight at day 19 post EL4 cells injection. **(D)** Percentage of mice with metastatic nodules in kidney or liver **(F)** was calculated. Also, number of metastatic nodules in kidney **(E)** or liver **(G)** was assessed. Values are expressed as mean ± SEM. *n* = 6 mice per group. *p < 0.05, **p < 0.01 respect to control mice.

For this purpose, irradiated mice were tail vein injected with lymphoid cells from control and stressed animals treated or not with fluoxetine or sertraline. After this procedure, mice were inoculated with tumor cells and the tumor growth and spontaneous metastasis were determined (see scheme in Figure 9A). A significant effect depending on time, stress exposure and SSRIs treatment of the injected cells was found (*F*_22,352_ = 5.207; *p* < 0.001). As it can be seen in Figure 9B, data indicated that tumor growth was increased after day 16 in mice injected with lymphocytes from chronically stressed mice when compared with those transferred with immune cells from control animals. Furthermore, mice injected with immune cells from stressed animals treated with fluoxetine and sertraline did not show this effect. Accordingly, tumor weight at day 18 post EL4 cells injection was significant depending on stress exposure and SSRIs treatment of the transferred cells (*F*_2,32_ = 3.586; *p* = 0.039) (Figure 9C). In addition, the assessment of spontaneous metastasis indicated that animals transferred with lymphoid cells from stressed animals had a higher incidence of liver metastasis (*p* = 0.023) (Figure 9D) and a major number of liver metastatic nodules (Figure 9E) respect to animals transferred with cells from control animals (*U* = 3.5, *p* = 0.027). These differences were not significant in kidney metastasis incidence (*p* = 0.740) (Figure 9F) or number of metastatic nodules (*U* = 7.5, *p* = 0.089) (Figure 9G). In addition, tumors from animals that were transferred with cells from stressed animals that had received fluoxetine or sertraline administration showed a similar biological behavior than those transferred with cells from control animals.

### Experiments Using Immune Cell-Depleted Tumor Cell Suspensions Demonstrate That Molecular Alterations Observed in the Total Tumor Mass Are Mainly due to Changes in the Cancer Cells

To further ascertain whether the alterations in cell cycle gene expression, cell migration, and MMPs expression described above were originated by the direct effect of treatment on cancer cells, or if the immune cells present in the tumor microenvironment were responsible for these changes, molecular alterations were assessed in immune cell-depleted tumor cell suspensions.

One-way ANOVA showed significant differences between groups for cyclin A2 (*F*_2,14_ = 7.483; *p* = 0.008), D1 (*F*_2,14_ = 6.519; *p* = 0.012), and p16 (*F*_2,14_ = 4.437; *p* = 0.036) expression levels. Results displayed in Table 2 indicate that mRNA levels of cyclins A2, D1 were significantly increased and p16 decreased in immune cell-depleted suspensions from animals under stress. Treatment with fluoxetine prevented these changes.

**Table 2:** Effect of chronic stress and fluoxetine treatment on molecular changes and migratory ability in immune cell-depleted tumor cells.

		C	S	S Fluoxetine
mRNA expression of cell cycle proteins	A2	0.999 ± 0.096	2.075 ± 0.376**	1.170 ± 0.117
	D1	0.996 ± 0,084	1.688 ± 0.177*	1.076 ± 0.166
	p16	1.080 ± 0.181	0.530 ± 0.114*	1.087 ± 0.151

mRNA expression of MMPs and TIMPs	MMP-2	1.124 ± 0.079	1.927 ± 0.147*	1.290 ± 0.277
	MMP-9	1.024 ± 0.204	2.414 ± 0.558*	1.032 ± 0.082
	TIMP-1	1.153 ± 0.247	0.341 ± 0.061**	1.110 ± 0.131

Cell migration (%)		5.649 ± 0.905	9.752 ± 1.331*	5.745 ± 0.756

Quantitative real-time reverse transcription polymerase chain reaction was performed in cDNA obtained from purified tumor cells suspensions from control (C), stressed (S), and fluoxetine-treated stressed (S Fluoxetine) mice. mRNA relative expression was quantified using β_2_-microglobulin as housekeeper. Moreover, isolated cells from solid tumors were seeded in the upper well of transwell migration chamber and RPMI with 10% FBS as attractant. After 24 h, cells in the upper and in the lower chamber were counted. Cell migration is presented as percentage of total cell count for each sample. Values are expressed as mean ± SEM. *n* = 5 mice per group. *p < 0.05, **p < 0.01 respect to control mice.

Similarly, significant differences between groups were found for MMP-2 (*F*_2,14_ = 5.151; *p* = 0.024), MMP-9 (*F*_2,14_ = 5.826; *p* = 0.017), and TIMP-1 (*F*_2,14_ = 7.627; *p* = 0.007) mRNA expression. *Post hoc* analysis indicated a significant increase in MMPs and a decrease in TIMP-1 levels induced by stress that were not observed in stressed animals treated with fluoxetine (Table 2).

In accordance with these results, one-way ANOVA of transwell migration assay data showed that the percentage of migration was significantly different depending on the treatment (*F*_2,14_ = 5.199; *p* = 0.024). *Post hoc* analysis indicated that immune cell-depleted suspensions from stressed animals have a greater migration capacity in a transwell chamber using FBS as attractant. Again, fluoxetine treatment impeded this effect (Table 2).

## Discussion

Epidemiologic and experimental animal research have indicated that stress may influence tumor progression (2–4, 7). However, the biological interactions between mediators of stress response, immune system, and tumor biology are not well understood. In particular, the role of the immune system in controlling solid tumor growth and dissemination has been considered unclear. In this context, our results showed a relevant role of antitumor immunity in solid tumor growth and in the invasion and dissemination of tumor cells. Our results indicated that chronic stress induces an alteration of immune homeostasis that in turn leads to an increase in both tumor growth and tumor cell dissemination. In addition, an important beneficious effect of fluoxetine or sertraline treatment was found.

Chronic stress exposure resulted in an increase of tumor growth. This effect was related to an enhancement of cell cycle progression through the modulation of cell cycle regulatory proteins. We observed an increase in the mRNA levels of cyclins A2, D1, and D3 in the tumors from stressed animals. A decrease in mRNA levels of cell cycle inhibitors p15/Ink4b, p16/Ink4a, p21/Cip1, and p27/Kip1 was also found. Much evidence have pointed the involvement of cyclins D1 and D3 in T-cell lymphomagenesis, and they have been highlighted as relevant molecular markers of oncogenic power in T cell lymphomas (38, 39). In addition, an increase of cyclin D3 has been linked to a high proliferation rate and with reduced levels of p27/Kip1 (40). Cyclins D1 and D3 upregulation has been related to a poor outcome in lymphoma bearing patients (41–43).

A robust correlation between a big tumor load, higher tumor growth, and increased chance of metastasis has been demonstrated in many human cancers (44, 45). In accordance with this association, our results showed an important increase of the incidence and number of spontaneous metastasis in stressed animals. In addition, we analyzed experimental metastases after intravenous injection into the lateral tail vein. Our results indicated that no differences were found in the incidence and number of nodules in the kidney and liver when control and stressed animals were injected with EL4 cells from culture. However, when untreated animals were injected with cells obtained from tumors that had been grown in stressed animals, the incidence and number of metastatic nodules were significantly higher than those obtained when injecting cells from tumors that had been dissected from control animals. These results indicate that stress modifies the capacity of cells to give metastatic colonization in distant tissues.

To metastasize, cancer cells have to migrate, overpass the extracellular matrix (ECM), invade blood vessels, adhere to a remote place, and extravasate to originate a distant foci. MMPs are a zinc-dependent endopeptidases family that are able of disrupting the main components of the ECM and that have a relevant role in pathological situations that course with a significant degradation of ECM, such as tumor invasion, and tumor metastasis (46). In addition, despite MMPs are expressed by multiple cell types in tumors, it has been probed that they exert broad pro-tumoral functions and their increase in tumors indicate a high-metastatic capacity (47). The action of MMPs is partially regulated by TIMPs, and the MMPs/TIMP activities balance is relevant for ECM turnover (48). Our results showed greater MMP-2 and MMP-9 and lower TIMP 1, 2, and 3 mRNA levels in tumors from stressed animals compared with control. Moreover, cells from tumors of stressed animals have a major ability to directionally respond to chemoattractants in the transwell migration assays.

It is important to consider that the determinations of cell cycle, MMP, and TIMP gene expression levels were performed using total tumor RNA. However, it is known that in the tumor microenvironment there is a complex variety of cells that express these genes. In particular, recent reports demonstrated that antigen-specific CD8+ tumor-infiltrating lymphocytes are actively proliferating, but also have a high apoptosis rate (49). Moreover, among the innate and adaptive immune cells recruited to the tumor site, macrophages are particularly abundant and are present at all stages of tumor progression (50). In this context, the stress-induced alterations observed in the whole tumor could be, at least in part, due to the immune cells from the tumor microenvironment. To ascertain if the molecular changes took place in cancer cells or in the infiltrating immune cells, we performed experiments using tumor cell suspensions depleted of the main infiltrating immune cell subsets, namely CD4+ and CD8+ T lymphocytes and macrophages (19, 50, 51). Our results showed that cell cycle gene expression changes induced by stress were similar in both, immune-depleted and not depleted tumor cell suspensions. However, a higher increase in MMP-2 expression levels was found in total tumor cells (% of increase, stressed vs control: 169) respect to immune cell-depleted suspensions (72%). Non-significant differences were observed in MMP-9 and TIMP-1 expression levels. Transwell assay results also indicated that the increased migration, induced by stress, of total tumor cells was higher than the observed for immune cell-depleted cell suspensions (160 vs 72%). Taken together, these results indicate that stress-induced tumor growth could be mainly related to molecular changes in cancer cells and that the greater invasive capacity of tumors from stressed animals is related to molecular changes in both cancer and tumor-infiltrating immune cells. Noteworthy, fluoxetine treatment reverted the effect of stress in both total tumor cell suspensions and immune cell-depleted suspensions.

Many findings have suggested a dynamic bidirectional dialogue between tumors and the immune system that modulates tumor growth and metastasis (52). The concept of cancer immunosurveillance (53) argues that cells of the innate and adaptive immune systems eliminate tumor cells thus protecting the host against tumor development. However, as cancer progress, tumor variants that are able to evade immune-mediated elimination appear and generate clinically apparent neoplasms. This evidence lead to a new assumption, the cancer immunoediting hypothesis, which emphasizes the dual role of immune system: host protective and tumor modeling on developing tumors (54).

Our results indicate that antitumor immunity was decreased in mice submitted to chronic stress. It could be possible to postulate that stress decreases immune response, thus favoring tumor growth. However, due to the unclear performance of the immune system in managing solid tumor progression under stress situations, the possibility that stress mediators, in particular the activation of the sympathetic nervous system, may straightly regulate the tumor behavior has been investigated (14, 55). Experimental analyses in animal models have found that behavioral stress induced an accelerated progression of pancreatic (56), prostate (57), breast (58), and ovarian (12) carcinomas and malignant melanomas (59). In addition, it was demonstrated that the biological action of stress could be effectively inhibited by β-adrenergic antagonists and simulated by β-agonists (11, 60, 61).

To elucidate if the effects of stress were due to a direct action of hormonal stress mediators on tumor cells or an indirect action through the alteration of the immune response, we performed an adoptive immune cell transfer experiment. Our results indicated that when irradiated animals were transferred with immune cells from stressed animals, a higher tumor growth and an increased number of spontaneous metastasis were observed compared with animals transferred with immune cells from control animals. It is important to note that irradiated animals were not submitted to stress in the whole experiment. These results indicated that, in our experimental model, the effect of stress on tumor progression was mediated mainly by immune cells.

Finally, our results indicate that fluoxetine or sertraline treatment were able to inhibit the effect of stress on tumor progression. Antidepressants are frequently used in cancer patients to treat their emotional disorders, such as depression and dysthymia. Nevertheless, clinical studies have not revealed clear effects of treatment with antidepressant in patients with cancer (62).

Nowadays, there are evidences that suggest that SSRIs could be useful in either treating cancer administered alone or in combination with standard chemotherapies (63). In addition, antidepressants, and more specifically SSRIs have been shown to reduce the risk of certain cancers (64–67). Moreover, these antidepressants have been shown to be oncolytic *in vitro* and *in vivo*, through a mechanism that involves an increase of the intracellular influx of Ca^2+^ (68–70) and/or a disruption of the mitochondrial membrane potential as well as the generation of reactive oxygen species (68, 71, 72). In general, these studies have been focalized on the direct action of SSRIs on tumor biology without taking into account the effect on antitumor immunity.

Our results emphasize the crucial role of the immune system in tumor progression under stress situations. Although a direct effect of stress and SSRIs treatment on tumor biology could not be ruled out, the beneficial effects of fluoxetine and sertraline appear to be mainly due to the restoration of the antitumor immune response. It is important to carry out investigations that allow to identify SSRIs targets outside their primary mechanism of action and thus encourage the performance of clinical studies leading to find significant benefits of the SSRIs prescription in cancer patients.

## Ethics Statement

This study was carried out in accordance with the recommendations of “Guide for the Care and Use of Laboratory Animals” (NIH) (revision 2011) and of the EC Directive 86/609/EEC (revision 2010). The protocol was approved by the local Institutional Committee for the Use and Care of Laboratory Animal rules (CICUAL, Instituto de Investigaciones Biomédicas-UCA-CONICET, Argentina).

## Author Contributions

GC and AG contributed to the conception of the work. MD and AG designed the study. MD performed the *in vivo* experiments. MD and HS carried out the *in vitro* experiments. MD and AG analyzed and interpreted the data and drafted the manuscript. HS and GC critically revised the manuscript. All the authors read and approved the final manuscript.

## Conflict of Interest Statement

The authors declare that the research was conducted in the absence of any commercial or financial relationships that could be construed as a potential conflict of interest.
